# Can change in phase angle predict the risk of morbidity and mortality during an 18-year follow-up period? A cohort study among adults

**DOI:** 10.3389/fnut.2023.1157531

**Published:** 2023-05-02

**Authors:** Raquel D. Langer, Leigh C. Ward, Sofus C. Larsen, Berit L. Heitmann

**Affiliations:** ^1^Research Unit for Dietary Studies, Parker Institute, Copenhagen, Denmark; ^2^Growth and Development Laboratory, Center for Investigation in Pediatrics, State University of Campinas, Campinas, Brazil; ^3^School of Chemistry and Molecular Biosciences, Faculty of Science, The University of Queensland, Brisbane, QLD, Australia; ^4^Section for General Practice, Department of Public Health, University of Copenhagen, Copenhagen, Denmark; ^5^The Boden Initiative, Charles Perkins Centre, The University of Sydney, Sydney, NSW, Australia

**Keywords:** bioelectrical impedance analysis, phase angle, nutritional status, mortality, cardiovascular disease

## Abstract

**Introduction:**

Phase angle (PhA, degrees), measured *via* bioimpedance (BIA, 50 kHz), is an index that has been used as an indicator of nutritional status and mortality in several clinical situations. This study aimed to determine the relationship between 6-year changes in PhA and total mortality as well as the risk of incident morbidity and mortality from cardiovascular disease (CVD) and coronary heart disease (CHD) during 18 years of follow-up among otherwise healthy adults.

**Methods:**

A random subset (*n* = 1,987) of 35–65 years old men and women was examined at the baseline in 1987/1988 and 6 years later in 1993/1994. Measures included weight, height, and whole-body BIA, from which PhA was calculated. Information on lifestyle was obtained through a questionnaire. The associations between 6-year PhA changes (ΔPhA) and incident CVD and CHD were assessed by Cox proportional hazard models. The median value of ΔPhA was used as the reference value. The hazard ratio (HR) model and confidence intervals (CIs) of incident CVD and CHD were used according to the 5th, 10th, 25th, 50th, 75th, 90th, and 95th percentiles of ΔPhA.

**Results:**

During 18 years of follow-up, 205 women and 289 men died. A higher risk of both total mortality and incident CVD was present below the 50th percentile (Δ = −0.85°). The highest risk was observed below the 5th percentile (ΔPhA = −2.60°) in relation to total mortality (HR: 1.55; 95% CI: 1.10–2.19) and incident CVD (HR: 1.52; 95% CI: 1.16–2.00).

**Discussion:**

The larger the decrease in PhA, the higher the risk of early mortality and incident CVD over the subsequent 18 years. PhA is a reliable and easy measure that may help identify those apparently healthy individuals who may be at increased risk of future CVD or dying prematurely. More studies are needed to confirm our results before it can be definitively concluded that PhA changes can improve clinical risk prediction.

## Introduction

Cardiovascular disease (CVD) is the leading risk factor for early mortality worldwide (1). In 2019, an estimated 17.9 million people died from CVD, representing 32% of all global deaths. Coronary heart disease (CHD) is the most common type of heart disease, killing ~360,900 people in 2019, and ~2 in 10 deaths from CHD happen in adults younger than 65 years old (2). While being physically inactive can increase the risk of myocardial infarction by >10%, adopting a generally healthy physically active lifestyle can further lower the risk of CVDs (3). Other behaviors such as cessation of tobacco use, reducing salt in the diet, eating more fruit and vegetables, and avoiding harmful alcohol use may also help decrease the risk of CVDs (1). During the development of CVD, subtle changes in body water may occur that, as suggested earlier, maybe the first sign of future increased risk of CVD (4), and changes, especially an increase in extracellular body water, as measured by bioelectrical impedance analysis (BIA), were suggested as a simple and easy to measure early prognostic marker to identify individuals in need of CVD prevention intervention (4).

Bioelectrical impedance analysis (BIA) is an indirect method used to estimate body composition, including body water, in a safe, fast, and non-invasive approach compared with other methods. Phase angle (PhA), determined by BIA at a frequency of 50 kHz (5), is an indicator of hydration and nutritional status as well as cell membrane integrity (6). PhA has been studied as a marker of morbidity and mortality in several diseases (7) and healthy populations (8). It has also been shown to be an indicator of general health status (9) and a prognostic survival factor in several diseases (hemodialysis patients, HIV infection, and cancers) (10–12). Low PhA (< 5.0°) indicates a compromised status of cell membranes and an imbalance of body water distribution, whereas a high PhA indicates better nutritional status and body cell mass (6, 13). Toso et al. (10) found a significant association between a low PhA (< 4.5°) and shorter survival in patients with advanced cancer. Thus, PhA can be used as a prognostic marker for morbidity and mortality in different clinical situations as well as among initially healthy individuals (7, 8).

While previous studies have used PhA to assess disease severity among patients or predict morbidity and mortality using a single time-point measure of PhA, it remains unknown if a change in PhA is also associated with an increased risk of morbidity and mortality among people free from primary chronic disease (8). Therefore, we aimed to determine the relationship between 6-year changes in PhA and 18-year incident morbidity and mortality from CVD and CHD in a random population sample of healthy adult Danish men and women. We hypothesized a reduction in PhA to be independently associated with an increased risk of morbidity and mortality from CVD and CHD, and that associations would be modified by sex and physical activity.

## Materials and methods

### Study population

**Figure 1:**
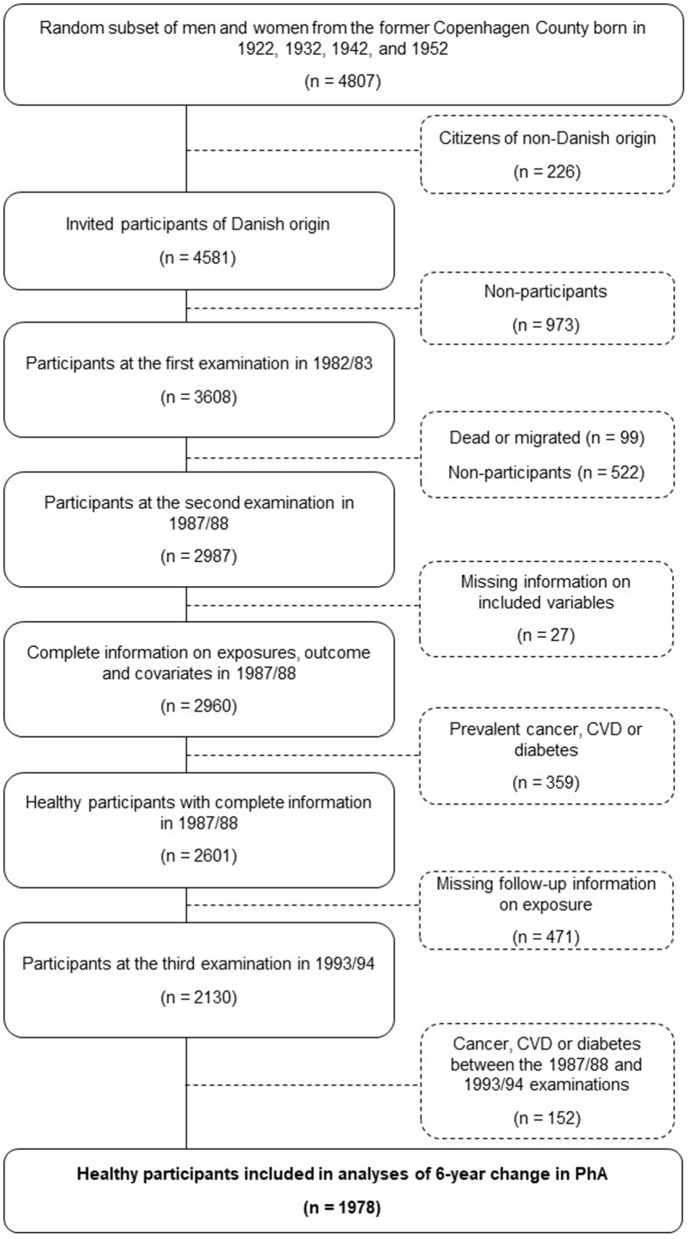
Flowchart of the study sample.

The study is part of the Danish Monitoring Trends in Cardiovascular Disease (MONICA) study, a longitudinal population-based study sampled among people from the greater Copenhagen area. Figure 1 shows the eligibility of the participants in the study, starting with the baseline MONICA cohort first examined in 1982/1983 as an age-stratified sample from subjects born in 1922, 1932, 1942, and 1952 selected randomly from the Danish Civil Registration System among individuals living in the greater Copenhagen area. The present study was based on measurements, including anthropometric and bioelectrical impedance, taken at the two follow-up surveys conducted in 1987/1988 and 1993/1994. Information about educational level, health behaviors, and prevalent major chronic diseases (cancer, CVD, or diabetes) was assessed *via* questionnaires and from record linkage to the National Patient Register (14). Participants who were of non-Danish origin were excluded, as well as all the participants who were diagnosed with cancer, CVD, or a self-reported diagnosis of diabetes before the 1993/1994 examination.

### Ethics statement

All participants gave written informed consent, and the ethical committee approved the study protocol of Copenhagen country. The study is under the Declaration of Helsinki for study with human subjects.

### Anthropometrics

Measurements for each participant were taken at the baseline (1987/1988) and after 6 years (1993/1994). Body weight (kg) was measured using a lever balance to the nearest 0.1 kg, and body height (cm) was measured using a wall-mounted stadiometer to the nearest 0.1 cm, following the recommended protocols from the World Health Organization (15).

### Bioelectrical impedance analysis

In 1987/1988, bioelectrical impedance measurements were taken on the right side of the body using a single frequency (50 kHz) BIA-103 RJL system analyzer (RJL Systems, Detroit, MI) and in 1993/1994 using the ImpediMed SEAC multi-frequency analyzer. Devices were calibrated against reference circuits and were technically accurate (16). Participants were overnight fasted and were instructed to remove all objects containing metal before measurement. All BIA measurements were taken with participants supine, with the arms and legs relaxed and slightly abducted from the body. Electrodes from the AccuSensor (Carbo Cone M45, Lynn Medical, Wixom, MI) were placed on the dorsal surfaces of the right hand and foot, at the distal metacarpals and metatarsals, respectively, and between the distal prominence of the radius and the ulna at the wrist and the medial and lateral malleoli at the ankle. The devices provided both the value of resistance (R) and reactance (Xc) in ohm (Ω), at 50 kHz, to calculate the PhA at this frequency in degrees as follows: PhA = arctangent (Xc/R) × (180°/π) (5).

### Endpoint

Information on incident CVD, CHD, and mortality was recovered from the Danish National Patient Registry, Cause of Death Register, and Central Person Register. Survival time was parameterized as the time until CVD (both fatal and non-fatal cases) or censoring event in the year, with age as the underlying time scale. Total CVD and CHD cases were identified based on International Classification of Disease codes 390 through 458 (8th edition) and 100 through 199 (10th edition). The subjects were followed until 4 October 2012, for an average of 18 years or a total of 46,831 persons per year.

### Covariates

Information on lifestyle factors was recovered through a detailed self-administered questionnaire at the baseline. Physical activity was categorized into sedentary (almost completely inactive, such as reading or watching television) and physically active [light activity at least 4 h/week (e.g., riding a bicycle or walking to work, walking, or skiing with the family, or gardening) or hard activity at least 3 h/week (e.g., heavy gardening, running, calisthenics, tennis, or regular hard physical training for competition, such as running events, soccer, racing, and European handball)] (17). Smoking behavior was defined as pack per year (20 cigarettes/pack) and computed as the product of smoking duration and cigarettes per day, divided by 20. Alcohol intake was defined as the number of standard drinks (12 g of alcohol each) per week. Both smoking and alcohol intake were included as continuous variables. The level of education was categorized into the primary level (< 10 grade) and the above primary level (>10 grade) of regular schooling.

### Statistical analysis

Characteristics of study participants were presented for men and women, according to mortality status as the median and corresponding interquartile range for continuous variables and as absolute numbers and percentages for categorical variables. Between-group differences were tested using the Wilcoxon rank-sum test or chi-square test. The associations between 6-year change in PhA and total mortality, incident CVD, and incident CHD older than 18 years were assessed using Cox proportional hazard regression models. Since the study cohort comprises four different birth cohorts with only a slight variation in age within the birth cohorts, time since follow-up examination was chosen as the underlying time scale instead of age. Both crude and adjusted models were conducted. The crude model was only adjusted for age and baseline level of PhA. The adjusted model was additionally adjusted for sex, weight, height, smoking, alcohol intake, physical activity, and level of education. The median value of a 6-year change in PhA was used as the reference value. To explore non-linear associations, all results were presented using restricted cubic splines with three knots placed at equally spaced percentiles of the exposure. All the reported plots represent the hazard ratio (HR) model as cubic spline functions for the 1st−99th percentile range of the exposure variable, avoiding not interpreting extreme values for which there were only limited data.

Effect modification between a 6-year change in PhA and sex or physical activity was examined by adding product terms to the model, and stratified analyses were conducted if statistically significant interaction was observed. The assumption of proportional hazards in the models was tested using Schoenfeld residuals.

All statistical tests were two-tailed with a significance level of *p* < 0.05. Analyses were performed using Stata SE 14 (StataCorp LLC, College Station, Texas, United States).

## Results

Table 1 presents the general characteristics of participants included in the study, stratified by sex and mortality. During follow-up, a total of 205 women and 289 men died. Women who died during follow-up had a higher incidence of CVD (67.3% vs. 35.0%) and CHD (16.6% vs. 8.0%), were older and shorter, had lower Xc and PhA values, and had a greater decrease in PhA over the 6-year study period than women who were censored (−0.9° vs. −0.8°, *p* = 0.006). They also had a lower educational level and smoked more compared to women who were censored (*p* < 0.001). Men who died during follow-up had a higher incidence of CVD (59.9% vs. 39.0%) and CHD (20.4% vs. 14.2%), were older and shorter, had a lower PhA values, and had a greater 6-year decrease in Xc and PhA (−1.1° vs. −0.8°, *p* < 0.001) values than men who were censored. They also were less physically active, had lower education levels, and smoked more compared to men who were censored (*p* < 0.001).

**Table 1:** Descriptive characteristics of the study participants at the 1987/1988 examination stratified by sex and mortality.

**Variables**	**Women**			**Men**		
	**Censored (*****n*** = **778)**	**Dead (*****n*** = **205)**	***p*** **-value**	**Censored (*****n*** = **547)**	**Dead (*****n*** = **289)**	***p*** **-value**
Age (years)	45.7 (35.9, 55.6)	65.5 (55.7, 65.9)	**< 0.001**	45.7 (35.9, 46.5)	56.0 (46.3, 65.7)	**< 0.001**
Weight (kg)	62.8 (57.7, 69.9)	63.3 (56.5, 71.9)	0.96	78.2 (71.5, 86.2)	78.6 (70.2, 87.3)	0.99
Height (cm)	164.0 (160.5, 168.0)	161.0 (157.5, 164.5)	**< 0.001**	177.0 (172.5, 181.5)	173.5 (170.0, 178.0)	**< 0.001**
R (Ω)	568 (527, 606)	565 (526, 610)	0.97	460 (432, 495)	464 (438, 501)	0.09
Δ R (Ω)	0.0 (−21.0, 20.0)	3.0 (−17.0, 23.0)	0.17	−1.0 (−14.0, 12.0)	2.0 (−14.0, 17.0)	0.05
Xc (Ω)	61.0 (56.0, 68.0)	58.0 (52.0, 66.0)	**< 0.001**	57.0 (52.0, 63.0)	57.0 (51.0, 64.0)	0.11
Δ Xc (Ω)	−8.0 (−14.0, −3.0)	−9.0 (−15.0, −4.0)	0.09	−7.0 (−12.0, −3.0)	−9.0 (−14.0, −4.0)	**< 0.001**
PhA (°)	6.2 (5.8, 6.8)	5.8 (5.4, 6.6)	**< 0.001**	7.1 (6.6, 7.7)	6.9 (6.2, 7.6)	**< 0.001**
Δ PhA (°)	−0.8 (−1.3, −0.3)	−0.9 (−1.4, −0.5)	**0.01**	−0.8 (−1.4, −0.4)	−1.1 (−1.8, −0.6)	**< 0.001**
CVD cases (%)	272 (35.0%)	138 (67.3%)	**< 0.001**	275 (39.0%)	173 (59.9%)	**< 0.001**
CHD cases (%)	62 (8.0%)	34 (16.6%)	**< 0.001**	100 (14.2%)	59 (20.4%)	**0.02**
**Physical activity level**, ***n*** **(%)**						
Sedentary	682 (87.7%)	181 (88.3%)	0.81	496 (70.3%)	236 (81.7%)	**< 0.001**
Active	96 (12.3%)	24 (11.7%)		210 (29.7%)	53 (18.3%)	
Smoking (pack/y)	3.8 (0.0, 15.8)	11.5 (0.0, 25.0)	**< 0.001**	11.0 (0.0, 23.8)	26.3 (10.5, 41.0)	**< 0.001**
Alcohol intake (units/wk)	3.0 (1.0, 7.0)	3.0 (0.0, 7.0)	0.21	8.0 (4.0, 17.0)	10.0 (4.0, 21.0)	0.06
**Educational level**, ***n*** **(%)**						
Primary	199 (25.6%)	96 (46.8%)	**< 0.001**	152 (21.5%)	135 (46.7%)	**< 0.001**
Beyond primary	579 (74.4%)	109 (53.2%)		554 (78.5%)	154 (53.3%)	

R, resistance; Xc, reactance; PhA, phase angle; Δ, 6-year change; CVD, cardiovascular disease; CHD, coronary heart disease. Results presented as median (interquartile range) unless otherwise stated. Bold values indicate statistically significant (censored vs. dead group), p < 0.05.

No evidence was found of an interaction between physical activity level and change in PhA on total mortality (*p* = 0.50), incident CVD (*p* = 0.27), or incident CHD (*p* = 0.50); moreover, no interaction between sex and change in PhA was found on total mortality (*p* = 0.96), incident CVD (*p* = 0.29), or incident CHD (*p* = 0.84). Thus, we did not stratify the analyses, but rather in addition to the baseline level of PhA and age, weight, height, smoking, alcohol intake, and level of education adjusted for both sex and physical activity in the fully adjusted regression models.

Table 2 shows crude and adjusted HRs (95% CIs) estimated from a Cox regression spline model of total mortality, incident CVD, and incident CHD, according to the 5th, 10th, 25th, 50th, 75th, 90th, and 95th percentiles of 6-years change in PhA (ΔPhA) among participants. After adjustment for covariates, a statistically significant association between a 6-year decrease in the PhA value (Δ = −0.85°) with the risk of total mortality and incident CVD was present below the 50th percentile (*p* < 0.001). The highest risk was observed below the 5th percentile (ΔPhA = −2.60°) in relation to total mortality (HR: 1.55; 95% CI: 1.10–2.19) and incident CVD (HR: 1.52; 95% CI: 1.16–2.00). We found no statistically significant association between a 6-year change in PhA and the risk of developing CHD.

**Table 2:** Hazard ratio (HR) model and confidence intervals (CIs)^*^ of total mortality, incident cardiovascular disease (CVD), and incident coronary heart disease (CHD) according to percentiles of 6-year change in phase angle (ΔPhA).

**Percentile**	**Δ PhA**	**Total mortality**						**CVD**						**CHD**					
		**Crude**			**Adjusted**			**Crude**			**Adjusted**			**Crude**			**Adjusted**		
		**HR**	**95% CI**		**HR**	**95% CI**		**HR**	**95% CI**		**HR**	**95% CI**		**HR**	**95% CI**		**HR**	**95% CI**	
5th	−2.60	1.10	0.80	1.52	1.55	1.10	2.19	1.30	1.00	1.68	1.52	1.16	2.00	0.83	0.51	1.33	1.29	0.77	2.16
10th	−2.06	1.06	0.86	1.31	1.34	1.08	1.67	1.19	1.01	1.41	1.32	1.11	1.58	0.90	0.66	1.23	1.22	0.87	1.70
25th	−1.39	1.02	0.94	1.11	1.12	1.03	1.22	1.07	1.01	1.14	1.12	1.05	1.20	0.99	0.88	1.12	1.12	0.98	1.28
50th	−0.85	1	1	1	1	1	1	1	1	1	1	1	1	1	1	1	1	1	1
75th	−0.41	1.00	0.91	1.09	0.94	0.86	1.02	0.96	0.90	1.02	0.93	0.87	1.00	0.94	0.82	1.08	0.87	0.76	1.00
90th	−0.07	1.00	0.84	1.19	0.90	0.77	1.06	0.93	0.82	1.06	0.89	0.78	1.02	0.87	0.67	1.14	0.77	0.58	1.00
95th	0.16	1.00	0.79	1.26	0.87	0.71	1.09	0.91	0.77	1.08	0.87	0.72	1.03	0.83	0.58	1.19	0.70	0.49	1.01

^*^Crude model was adjusted for age and baseline level of PhA. The adjusted model was additionally adjusted for sex, weight, height, smoking, alcohol intake, physical activity, and level of education.

**Figure 2:**
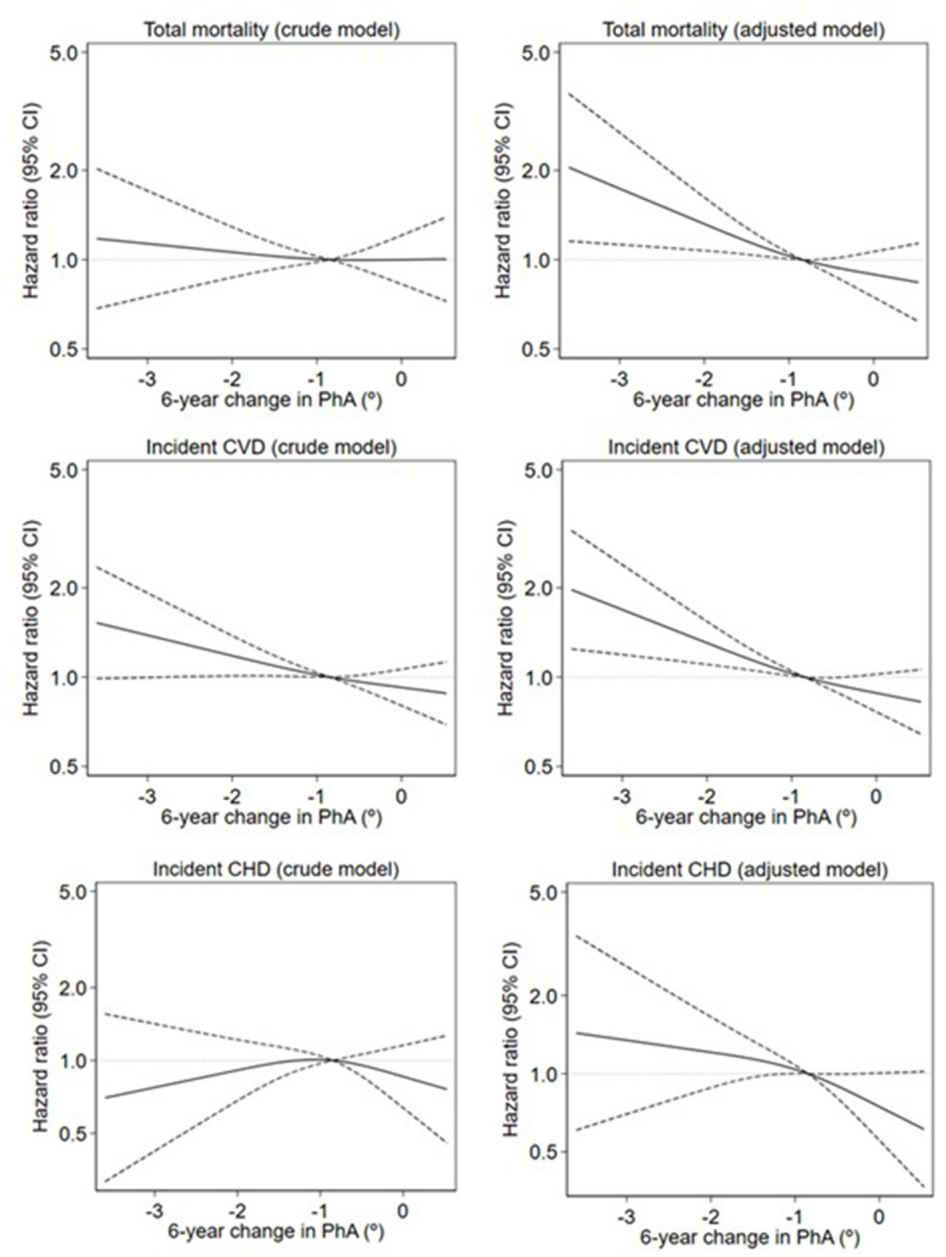
Crude and adjusted restricted cubic splines of the association between 6-year change in phase angle (PhA) and total mortality, incident cardiovascular disease (CVD), and incident coronary heart disease (CHD). Mortality is estimated by hazard ratios (smoothed lines) with 95% confidence intervals (dashed lines) estimated from a Cox regression spline model. The crude models were adjusted only for age and baseline level of PhA. The adjusted models were additionally adjusted for weight, height, smoking, alcohol intake, physical activity, and level of education. The median value of change in PhA is the reference value (hazard ratios of 1.00, indicated by the horizontal dotted line). Only the 1st−99th percentile of exposure is shown.

Figure 2 illustrates the crude and adjusted restricted cubic splines of the association between a 6-year change in PhA and total mortality, incident CVD, and incident CHD. A threshold effect of a 6-year change in PhA on total mortality and incident CVD was evident at around a decrease in PhA of −0.85°. Below this threshold, a higher risk of total mortality and incident CVD was observed, while no further risk reduction was observed above ΔPhA= −0.85°. A similar but not significant pattern of a threshold was seen for the association between the 6-year change in PhA and incident CHD.

## Discussion

This study aimed to determine the relationship between 6-year changes in PhA and subsequent 18-year total mortality or incident CVD and CHD among initially healthy (free from primary chronic disease) adult Danish men and women. Our findings indicated that men and women who were older than 6 years decreased the value of PhA by more than 1° had an increased risk of total mortality and incident CVD. An association between a low value of PhA and mortality risk has been shown previously among individuals with and without heart diseases (18–20) and hospitalized patients (21–23). However, to the best of our knowledge, no previous studies have examined if a measured change over time in PhA may be predictive of a future increased risk of early overall mortality or cardiovascular disease. Our results suggest that changes in PhA occurring almost 20 years earlier seem predictive of future mortality and CVD risk.

The present study builds on our previous findings in which a lower PhA value was associated with a higher incidence of CVD over 24 years in a group of adult Danish men and women free from primary chronic disease (8). In this previous study, among women, a higher risk of incident CVD was found when PhA was < 6.6°; however, among men, no significant association was found. It is well known that sex, age, and physical activity are some of the main factors to influence the PhA value (24, 25); however, in the present study, the morbidity and mortality risk related to changes in PhA was significant and not different among men and women. In addition, we found no evidence that associations between PhA changes and subsequent morbidity and mortality risk were different for subjects reporting high and non-daily physical activity. However, in a previous study with young healthy adults (26), army cadets (27), and older women (28), after a period of physical training, participants have shown an increase in the value of PhA. It is speculated that since participants included in the present study did not have a higher level of physical activity (compared to other studies), this may be a factor that did not influence the relationship between PhA and risk of CVD and mortality. The present findings show that changes in PhA were associated with an increased risk of total mortality and incident CVD, suggesting that PhA may be used as an important early prognostic tool for the survival and prevention of CVD.

A decrease in PhA would indicate that cell membranes may have become more compromised leading to an imbalance of body water distribution and potential accumulation in extracellular water, imbalances that may not be clinically visible or otherwise discovered (29, 30). In accordance, previous studies have reported that an excess of extracellular water was associated with the development of cardiovascular morbidity and mortality among older adults (4, 18, 19). In the present study, for most individuals, PhA decreased over the 6-year observation period; however, the increased risk of early mortality and CVD morbidity was mainly present below, but not above the 50th percentile, indicating a threshold effect of loss of PhA below which the risk of developing CVD and dying prematurely increased with a greater decrease in the PhA value (Δ = −0.85°). The highest risk was observed below the 5th percentile (ΔPhA = −2.60°) in relation to total mortality (HR: 1.55; 95% CI: 1.10–2.19) and incident CVD (HR: 1.52; 95% CI: 1.16–2.00). We found no statistically significant association between a 6-year change in PhA and the risk of developing CHD, possibly related to the lower number of individuals developing CHD than developing CVD.

The main strength of this study was that our sample included participants who were initially free of primary chronic disease and were followed for almost two decades for the development of premature mortality and incident CVD, providing a unique opportunity to investigate our hypothesis. Moreover, the Danish MONICA study had a very high participation rate (78.8%) (31). In addition, we have no reason to believe that the associations we have found would be different in other Western populations of generally Caucasian origin. Hence, our results may suggest a good model of health behaviors apply to other similar populations.

A potential limitation of our study is that the information on general lifestyle factors was obtained by a self-questionnaire, which, however, would have led to attenuation rather than inflation of the association between changes in PhA and CVD risk but may have masked potential significant associations between PhA change and CHD. Although we adjusted our analyses for several potential confounders, as in most observational studies, it is still likely that some unmeasured or residual confounding remained. As an example, our analyses were not adjusted for any measure of diet quality, which is a known risk factor for CVD and a predictor for mortality (32).

Our findings suggest that a decrease in PhA value over a 6-year period of more than 0.85° was associated with an increased subsequent risk of early mortality and incident CVD. This safe, reliable, and easily obtainable measure of PhA from BIA, which is indicative of changes in cell membrane health and fluid balance, may help identify those otherwise healthy (free from primary chronic disease) adults who are at an increased risk of future incident CVD or dying prematurely. However, to the best of our knowledge, this study is the first to examine if changes in PhA are predictive of future CVD morbidity and total mortality, more studies are still needed before it can be firmly concluded that PhA changes can improve clinical risk prediction.

## Data availability statement

Data from the Monica study cannot be made publicly available for ethical and legal reasons. Public availability may compromise participant privacy, and this would not comply with Danish legislation. Access to the data requires an application submitted to and subsequently approved by the steering committee. Contact BH (Berit.Lilienthal.Heitmann@regionh.dk) or the Research Unit for Dietary Studies at The Parker Institute (bfh-eek@regionh.dk).

## Ethics statement

All participants gave written informed consent, and the study was approved by the Local Ethics Committee of Copenhagen County. The study was conducted in accordance with the Helsinki Declaration.

## Author contributions

RL, LW, and BH designed the study. BH carried out data collection, provided all needed materials, and funding acquisition to conduct the study. SL performed the statistical analysis, analyzed the results, and created the figures. LW helped to analyze the impedance data. RL together with LW and BH drafted the manuscript. LW, BH, and SL revised the manuscript and added their valuable expertise to the discussion section. All authors approved the final version of the manuscript.
